# Meta-analysis of IDH-mutant cancers identifies EBF1 as an interaction partner for TET2

**DOI:** 10.1038/ncomms3166

**Published:** 2013-07-18

**Authors:** Paul Guilhamon, Malihe Eskandarpour, Dina Halai, Gareth A. Wilson, Andrew Feber, Andrew E. Teschendorff, Valenti Gomez, Alexander Hergovich, Roberto Tirabosco, M. Fernanda Amary, Daniel Baumhoer, Gernot Jundt, Mark T. Ross, Adrienne M. Flanagan, Stephan Beck

**Affiliations:** 1 Medical Genomics, UCL Cancer Institute, University College London, London, UK; 2 Genetics and Cell Biology of Sarcoma, UCL Cancer Institute, University College London, London, UK; 3 Department of Histopathology, Royal National Orthopaedic Hospital NHS Trust, Middlesex, UK; 4 Statistical Cancer Genomics, UCL Cancer Institute, University College London, London, UK; 5 Tumour Suppressor Signalling Networks, UCL Cancer Institute, University College London, London, UK; 6 Bone Tumor Reference Center at the Institute of Pathology, University Hospital Basel, Basel, Switzerland; 7 Illumina Cambridge Ltd., Chesterford Research Park, Little Chesterford, UK

## Abstract

Isocitrate dehydrogenase (*IDH*) genes 1 and 2 are frequently mutated in acute myeloid leukaemia (AML), low-grade glioma, cholangiocarcinoma (CC) and chondrosarcoma (CS). For AML, low-grade glioma and CC, mutant IDH status is associated with a DNA hypermethylation phenotype, implicating altered epigenome dynamics in the aetiology of these cancers. Here we show that the IDH variants in CS are also associated with a hypermethylation phenotype and display increased production of the oncometabolite 2-hydroxyglutarate, supporting the role of mutant IDH-produced 2-hydroxyglutarate as an inhibitor of TET-mediated DNA demethylation. Meta-analysis of the acute myeloid leukaemia, low-grade glioma, cholangiocarcinoma and CS methylation data identifies cancer-specific effectors within the retinoic acid receptor activation pathway among the hypermethylated targets. By analysing sequence motifs surrounding hypermethylated sites across the four cancer types, and using chromatin immunoprecipitation and western blotting, we identify the transcription factor EBF1 (early B-cell factor 1) as an interaction partner for TET2, suggesting a sequence-specific mechanism for regulating DNA methylation.

## Abstract (web summary)

Cancer-associated mutations in isocitrate dehydrogenase are proposed to impair TET2-dependent DNA demethylation. By comparing the methylomes of *IDH*-mutant cancers, the authors identify the transcription factor EBF1 as a partner of TET2, suggesting a possible means for targeting TET2 to specific DNA sequences.

## Graphical abstract

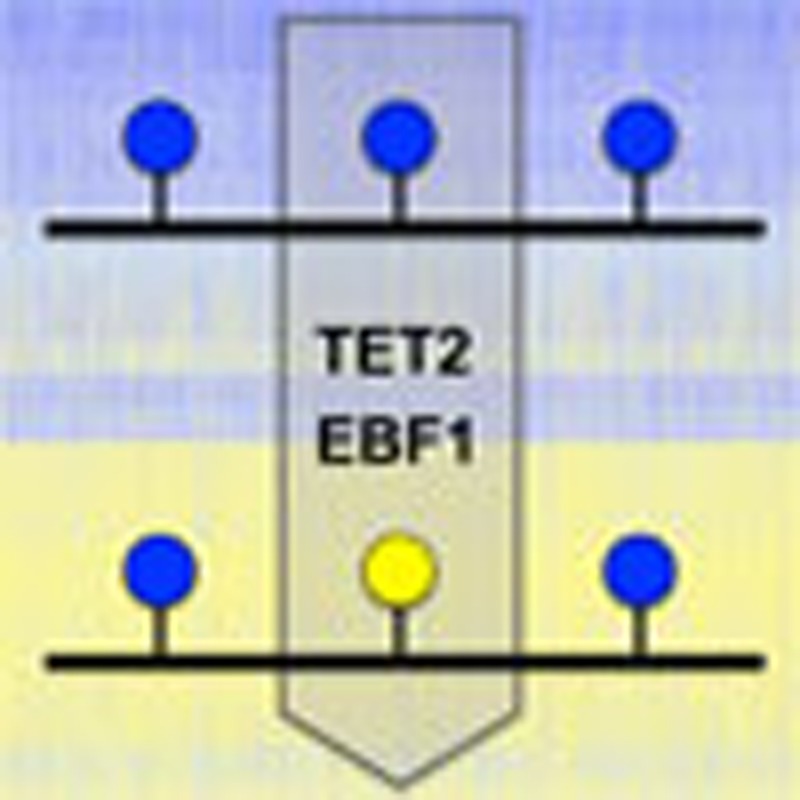

Chondrosarcoma (CS) is the second most common primary malignant bone tumour^1^. When such tumours arise as solitary lesions in the medullary cavity (central CS) or more rarely in the periosteum, ~50% harbour either a somatic *IDH1* (isocitrate dehydrogenase 1) or *IDH2* heterozygous mutation^2^. In a minority of individuals, these tumours are multiple, and affected individuals are at risk of developing other neoplasms, including spindle cell haemangiomas, and high-grade gliomas/secondary glioblastomas, among others^3^. In this setting, the mosaic distribution of tumours is caused by somatic early post-zygotic mutations of *IDH1* and *IDH2* (ref. 3). The same mutations have been previously identified in ~70% of sporadic high-grade gliomas and secondary glioblastomas^4^, ~10% of acute myeloid leukemias (AMLs)^4^ and cholangiocarcinomas (CCs)^5^, and much less commonly in other neoplasms. The mutant (mt) IDH enzyme catalyses the reduction of α-ketoglutarate (α-KG) to D-2-hydroxyglutarate (2-HG), an oncometabolite affecting the activity of α-KG–dependent dioxygenases^6^: these events affect a number of cellular responses, and have been shown to induce CpG island DNA hypermethylation in low-grade gliomas (LGGs)^7^, CCs^5^ and AMLs^8^ harbouring *IDH1* and *IDH2* mutations. The TET dioxygenases are responsible for the conversion of 5-methylcytosine to 5-hydroxymethylcytosine (5hmC)^9^, an intermediate metabolite in the recently discovered active demethylation pathway^10^,^11^, and it is possible that mt IDH1 enzyme mediates the observed hypermethylation phenotype through inhibition of TET by 2-HG^8^.

The evidence supporting the concept that the *IDH1* and *IDH2* mutations occur early in the genesis of these *IDH*-mt tumours^3^ suggests that the different neoplasms share a major regulatory effector. In this study, we aimed to identify the shared and tissue-specific processes by profiling the methylome of central CS with and without *IDH* mutations, and performing a meta-analysis of the publically available data sets from LGG, AML, CC and our CS data.

## Results

### DNA methylation analysis

**Figure 1:**
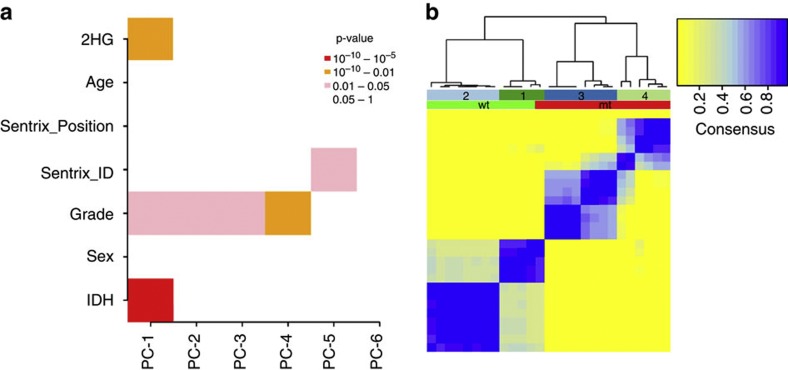
Principal component and unsupervised analysis of IDH mt-associated MVPs. (**a**) Singular value decomposition: PC-k refers to the k^th^ principal component; the first two components associate most significantly with *IDH* status (Kruskal–Wallis *P*-value=2 × 10^−6^) and 2-HG levels (*P*-value=6.8 × 10^−4^), while other significant components associate mainly with tumour grade. Technical component Sentrix ID is not correlated with variation in the data until PC-5. Other biological factors, such as patient age and sex are not correlated with any significant component. (**b**) Unsupervised consensus clustering of the top 150 MVPs across all samples as determined by median absolute deviation. Clusters one and two (left) are mainly populated by *IDH* wt samples (light green) with one mt included, while clusters three and four (right) are exclusively mt (red).

**Figure 2:**
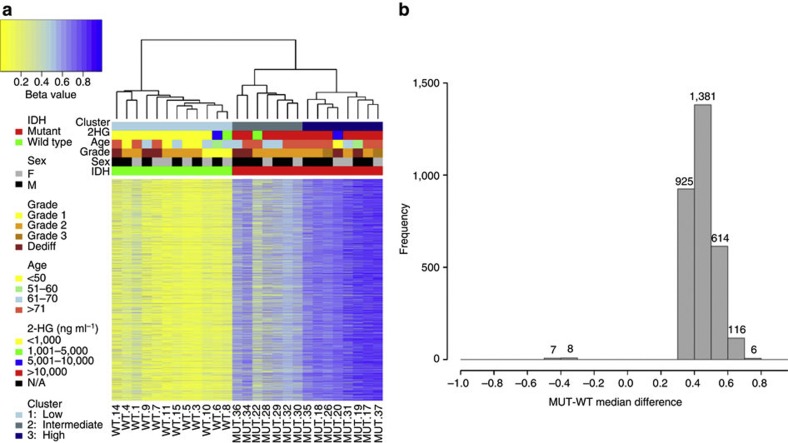
Supervised analysis reveals a hypermethylation phenotype associated with *IDH* mutation. (**a**) Hierarchical clustering of the top 3,057 hyper and hypo-MVPs (Wilcoxon rank-sum test *P*≤0.001 and |Δß|≥0.35) between *IDH* mt and wt. The samples cluster into three groups: low/unmethylated wt cluster (1) intermediate/high methylation mt cluster (2) highly methylated mt cluster (3) 2-HG levels positively correlate with *IDH* mutation and hypermethylation. (**b**) Frequency distribution of median ß-value differences between mt and wt sample groups in selected (top 3,057) probes. 99.5% (3,042 of 3,057) are hypermethylated in mt samples relative to wt.

To investigate the effects of *IDH1* and *IDH2* mutations on the CS methylome, we conducted genome-wide DNA methylation (DNAm) profiling of 44 central CS tumour samples using the Illumina Infinium 450 K BeadChips^12^. Following strict quality control (Methods), a final data set of 472,655 β values from 12 *IDH* wild-type (wt) and 15 *IDH*-mt samples was analysed. As defined previously^13^, probes or CpG sites are referred to as methylation variable positions (MVPs) and as hyper- or hypo-MVPs when directionality towards differential hyper- or hypomethylation has been ascertained. To determine the nature of the largest sources of variation in the data, we performed a principal component analysis (Fig. 1a). The top component was highly correlated with *IDH* mt status and 2-HG levels (Kruskal–Wallis *P*-value=2 × 10^−6^ and 6.8 × 10^−4^, respectively). We note that the association of both *IDH* mt status and 2-HG levels with the same principal component is not surprising given that *IDH* mt status and 2-HG levels were strongly correlated (Spearman rank correlation coefficient ρ=0.84; *P*-value=3.62 × 10^−8^). Tumour grade also appeared to have an impact on the first component, but this may be accounted for by the uneven distribution of the tumour grades in the two groups, with grade I and III tumours only being represented in the wt or mt groups, respectively (Fig. 2a). This appears to have occurred by chance, as our previous studies with larger numbers of cases found that there was no association of grade with mutation status^2^,^3^. No association was found with age of presentation and sex, and technical factors were also not (Sentrix Position) or only weakly (Sentrix ID) associated with principal components. Unsupervised consensus clustering (Fig. 1b) of the top 150 MVPs across all samples, as determined by the median absolute deviation estimator, supported *IDH* mt status as the main driver of changes in the DNA methylation: our sample cohort clustered into four groups, the first two of which were 92% *IDH* wt (12 wt and 1 mt), while the other two exclusively contained mt samples.

We then performed a supervised analysis using a Wilcoxon rank-sum test to determine the directionality of the MVPs between CS with and without *IDH* mutation. MVPs were selected on the basis of statistical significance (Wilcoxon *P*-value≤0.001), and an additional filter of |Δß|≥0.35 was applied to compensate for the Wilcoxon rank-sum test not taking into account the absolute difference in methylation between the groups, and to narrow down our search to differences with higher potential for functional effect. A total of 3,057 MVPs met these requirements, and hierarchical clustering of the samples yielded three distinct groups (Fig. 2a): a mt cluster defined by high hyper-MVPs (median β=0.75), a second mt cluster defined by intermediate to high hyper-MVPs (median β=0.55), and a wt cluster corresponding to hypo-MVPs (median β=0.16). Moreover, of the 3,057 MVPs between mt and wt CS, 99.5% (3,042/3,057) were hyper-MVPs in the mt group relative to the wt group, thereby revealing a strong hypermethylation phenotype associated with mutation(s) in the *IDH* genes in central CS (Fig. 2b).

**Figure 3:**
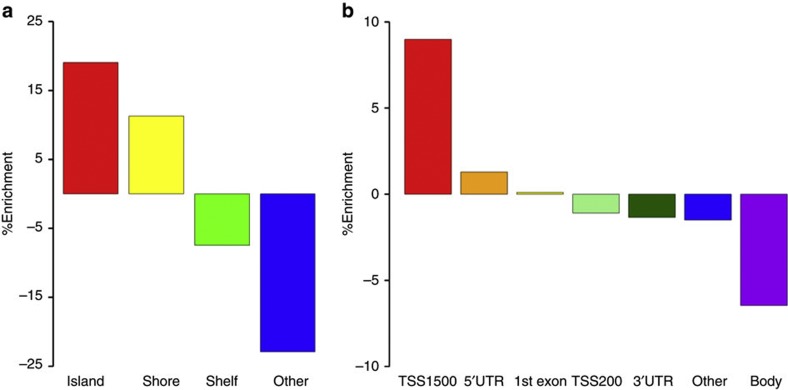
IDH mt-associated MVPs are enriched in CpG islands and gene promoters. (**a**) Percentage enrichment of epigenomic features (random resampling *P*-value≤0.001) as annotated on the Infinium 450 K BeadChip array: CpG Islands are defined as DNA sequences with GC content ≥50% and a CpG observed/expected ratio ≥0.6; CpG Shores correspond to the 2 kb sequences directly upstream and downstream of a CpG Island. Enrichment was assessed by repeated random sampling. Islands and shores are enriched for by 19.1 and 11.3%, respectively. (**b**) Percentage enrichment of genomic features (*P*-value≤0.001). TSS1500=1,500 bp upstream of transcription start site; TSS200=200 bp upstream of transcription start site; the TSS1500 region is enriched for by 9%, while probes located within the gene body are depleted by 6.4%.

Mapping of the hyper-MVPs to gene features revealed significant (random resampling *P*-value≤0.001) enrichment for regulatory regions (Fig. 3a) such as promoter-associated transcription start sites (9% for TSS1500) and CpG islands and shores, which were enriched by 19.1 and 11.3%, respectively. These regions are known to be of particular relevance to transcriptional regulation^14^.

### Validation and replication

For validation of results from the original data set, we additionally analysed 16 of the CS samples (5 wt and 11 mt) using two independent methods: pyrosequencing, and a novel high-throughput targeted technology based on microdroplet PCR coupled with next-generation sequencing (RainDrop-BSseq). For the latter, we targeted 855 CpG sites and validated their methylation status for 98.8% (426/431) and 95.5% (429/449) in the wt and mt groups, respectively (Supplementary Fig. S1a). For two of these sites, we also performed pyrosequencing in two wt and two mt samples and compared the results to the RainDrop-BSseq as well as the original 450 K array data. Results from all the three platforms agreed in every case, with a mean cross-platform difference of β=0.09 (min=0.01, max=0.19) (Supplementary Fig. S1a). Using RainDrop-BSseq, we further replicated the results for 373 sites in an independent set of six mt CS samples, and found matching methylation states in 94.3% (352/373) of cases (Supplementary Fig. S1b). Finally, an additional independent panel of 24 central CS (10 wt and 14 mt), analysed on the 450 K arrays, was used for further replication of the identified hypermethylation profile: the top 500 MVPs from the initial test set correctly separated the replication set into wt and mt clusters (22/24, 92%) (Supplementary Fig. S2a), with a clear hypermethylation profile in the mt group (Supplementary Fig. S2b).

### Meta-analysis

DNA methylation profiles of tumours with *IDH* mutations have also been studied in the context of three other malignancies: AML^8^, LGG^7^ and CC^5^. In all the three tumour types, a hypermethylation phenotype similar to that described for central CS was identified, suggesting a common mechanism linking gain of function in the IDH enzyme associated with reduced demethylation, possibly through inhibition of the TET family of oxygenases^15^. To determine whether this particular mechanism affects shared pathways and/or tissue-specific processes in each cancer type, we performed a meta-analysis using publically available methylation profiles from AML, LGG and CC (accession codes GSE24505, GSE30339 and GSE32286, respectively) and our own CS data (GSE40853). The analysis was conducted at gene level using IPA (Ingenuity Systems, www.ingenuity.com) (Methods).

The AML data set (*n*=398) was derived using the HELP^16^ (HpaII tiny fragment Enrichment by Ligation-mediated PCR) assay, which targets CpG sites in gene promoters. Both the publically available LGG (*n*=81) and CC (*n*=50) data sets, as well as our CS data were obtained from Illumina BeadChip 450 K methylation arrays, covering not only promoter regions of known genes but also gene bodies and certain intergenic sites. This allowed us to apply the same filters to the LGG and CC data as we employed for the supervised analysis of CS described above (Wilcoxon *P*-value≤0.001, |Δß|≥0.35). In order to facilitate inclusion into our meta-analysis of the AML data, restricted to gene promoters by the HELP assay, we then further refined the 450 K data sets to sites annotated to CpG islands or shores within promoter regions. Using this stratification, a total of 640, 1,028, 169 and 48 genes were available for comparison from CS, LGG, CC and AML, respectively. The CS and LGG data sets had 188 genes in common (random resampling *P*-value≤10^−5^), while CS, LGG and CC overlapped by 16 (random resampling *P*-value≤10^−6^), but no gene was found to be present in all four gene lists.

**Figure 4:**
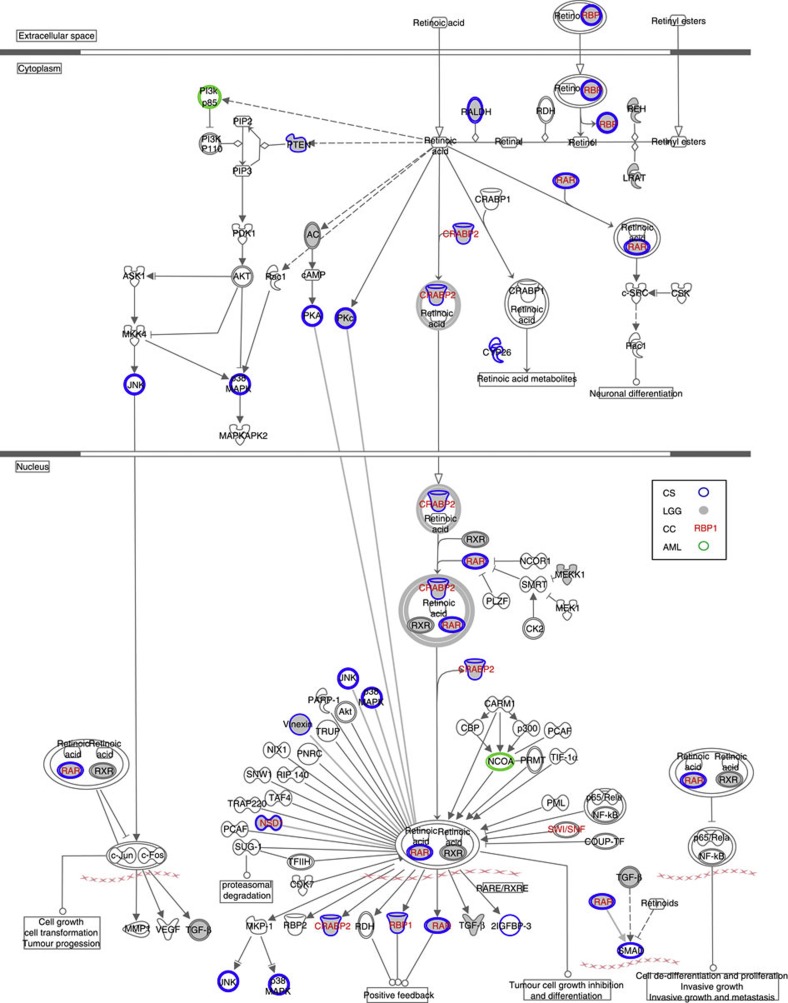
The RAR activation pathway is affected by differential methylation in *IDH*-mt cancers. Schematic diagram of the RAR activation pathway derived using IPA. Molecules are represented as nodes, and the biological relationship between two nodes is represented as an edge (line). All edges are supported by at least one reference from the literature, from a textbook, or from canonical information stored in the Ingenuity Knowledge Base. Nodes are displayed using various shapes that represent the functional class of the gene product. Edges are displayed with various labels that describe the nature of the relationship between the nodes (for example, P for phosphorylation, T for transcription).

We then analysed all genes from the four data sets for shared pathways, and although no canonical pathway as annotated by IPA reached statistical significance, retinoic acid receptor (RAR) activation was affected in all four cancer types, involving five genes from CC, two from AML, 17 from LGG and 14 genes from CS (Fig. 4). Retinol-binding protein 1, for example, was differentially methylated in CS, LGG and CC, and found in our cohort to display significant hypermethylation in the promoter region and downregulation of gene expression (Supplementary Fig. S3). However, in the four cancer types, the other top pathways were more directly related to each of the affected tissues: these included pathways involving the function of osteoblasts, osteoclasts and chondrocytes in CS, axonal guidance signalling in LGG, Myc signalling in AML, and circadian rhythm signalling in CC. This suggests a tissue-specific hypermethylation phenotype of each individual tumour type.

To investigate the findings further, we specifically analysed those genes that were uniquely differentially methylated in each cancer type. In CS, the most significantly affected physiological function category was tissue development (right-tailed Fisher exact test *P*-value=5.44 × 10^−5^−4.76 × 10^−2^; number of genes *n*=46), with development of connective tissue and adhesion of carcinoma cell lines and fibroblasts as the top functions. In LGG, the most significant category was nervous system development and function (*P*-value=9.43 × 10^−4^−4.38 × 10^−2^, *n*=12) with the extension of neurites and axons and the proliferation of neuronal cells as top function; moreover, the most significant diseases corresponding to the analysed gene set were hereditary disorders, neurological disease and psychological disorders (*P*-value=2.04 × 10^−5^−4.38 × 10^−2^). The top category in CC, an epithelial cell malignancy, was hair and skin development (*P*-value=2.85 × 10^−3^−2.63 × 10^−2^), with proliferation of epithelial cells as top function. Finally, we identified haematological disease (*P*-value=2.34 × 10^−3^−4.67 × 10^−3^) as the most significant disease in the AML data set.

**Figure 5:**
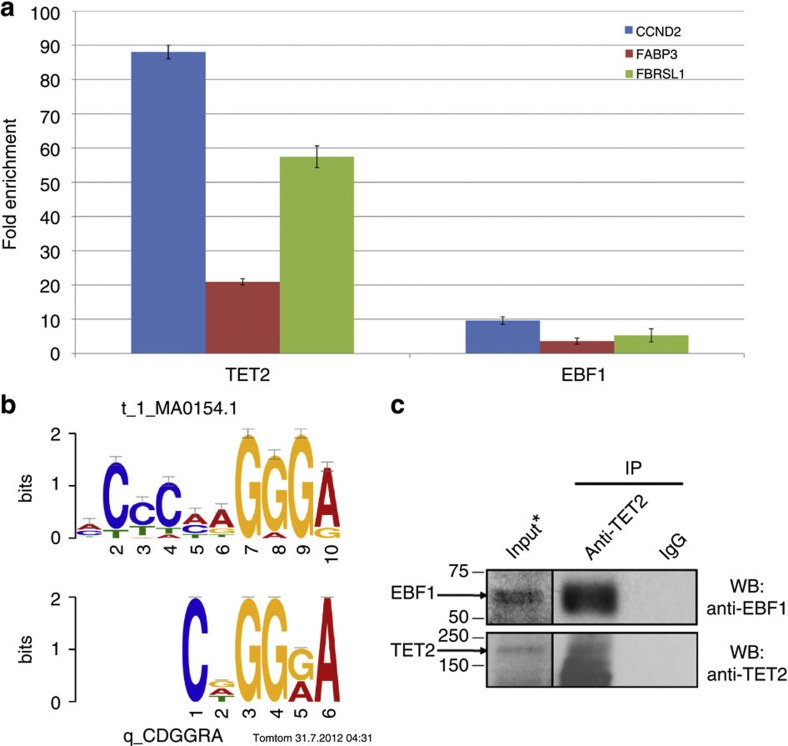
EBF1 interacts with TET2 at hypermethylated loci. (**a**) ChIP-qPCR analysis at three target sites shows proportional presence of both TET2 and EBF1 at these loci. Fold enrichments were calculated by ΔΔCt, normalising to both the mock IgG IP control and a negative control region where no binding of either protein was expected. Error bars correspond to standard errors of the mean, *n*=3. (**b**) Motif logo matching between CDGGRA (bottom) and the consensus EBF1 motif (top), as determined by TOMTOM (MEME). The offset of the sequence relative to the known motif was used in conjunction with the nucleotide frequencies in each motif to determine the significance of the match: TOMTOM *P*-value=0.00249671. (**c**) Endogenous EBF1 interacts with TET2 in SW1353 cells. Western blotting of anti-TET2 and control IgG immuno-complexes with indicated antibodies. A longer exposure of input lysates (5% of total) is indicated by an asterisk *.

A potential mechanism by which TET regulates demethylation in a tissue-specific manner could be through interaction with a DNA-binding partner. To test this hypothesis, we searched for common motifs in 100 bp windows surrounding the identified MVPs in CS, LGG and CC using the multiple expectation maximization for motif elicitation (MEME)^17^ suite and identified the 5′-CDGGRA-3′ motif as highly significant (MEME *P*-value=10^−3^, discriminative DNA motif discovery (DREME) *P*-value=7 × 10^−70^). The presence of this motif was then assessed over a 1 kb window around each MVP, as DNA methylation is known to be tightly correlated over that distance^18^,^19^, and the motif was found in 93% of sequences tested (8,008/8,582); it is also present in 73% of the AML sequences identified as differentially methylated. Using the TOMTOM tool within the MEME suite to test for similarity to known DNA-binding sites, we identified the early B-cell factor 1 (EBF1) binding motif as a significant match (TOMTOM *P*-value=0.0025) (Fig. 5a). To assess this prediction, we analysed publically available EBF1 chromatin immunoprecipitation sequencing (ChIP-seq) data from the UCSC Genome Browser and found the CDGGRA motif to be present in 40% of EBF1-enriched sequences. Additionally, we assessed the expression levels of EBF1 in a set of 32 CS samples (19 mt and 13 wt) and found the gene to be expressed at similar levels (Wilcoxon *P*-value=0.34) in both sample groups (Supplementary Fig. S4).

To validate the predicted interaction between TET2 and EBF1, we then conducted ChIP experiments in the SW1353 CS cell line with antibodies against TET2 and EBF1, and measured enrichment for these on three loci (*CCND2*, *FABP3* and *FBRSL1*). The targets were selected on the basis that they were significantly hypermethylated in our *IDH* mt CS cohort as compared to the wt samples, displayed high methylation (β value>0.9) in the SW1353 cell line, and contained at least one predicted binding site for EBF1 in a 100 bp window (Supplementary Table S2). A negative control region, to which the enrichments were normalised, was selected for being highly methylated in all samples, irrespective of the IDH mutation status, and having no EBF1 binding sites in its vicinity. All three target sites were enriched for TET2 and EBF1 (Fig. 5b) with fold enrichments ranging from 3.6 to 88. It is noteworthy that the TET2:EBF1 fold enrichment ratios were similar at the three sites (9.2, 5.8, and 10.1) further supporting co-localisation of the two proteins. Finally, in order to address the potential protein–protein interaction between TET2 and EBF1, we performed co-immunoprecipitation experiments using an antibody against TET2 (Fig. 5c). Significantly, EBF1 was detected by western blotting in the TET2 precipitate, demonstrating that endogenous TET2 and EBF1 interact in SW1353 cells.

## Discussion

The effects of gain-of-function mutations in the *IDH1/2* enzymes on the methylome have been extensively studied in the context of AML and LGG, and more recently in CC^5^,^7^,^8^. Here, we report methylation profiling data supporting a similar mechanism in central CS, where specific *IDH* mutations are correlated with increased levels of the 2-HG oncometabolite^3^ compared with wt tumours, and a widespread hypermethylation phenotype. While many epigenetic effector proteins, such as DNA methyl-transferases or histone deacetylases, are known to be mutated in human cancers, thus directly affecting tumour development^20^, the indirect inhibition of demethylation via *IDH* mutation suggests a new role for metabolism in the regulation of the epigenome. The genomic and epigenomic targets of the observed hypermethylation, focused mainly on CpG islands and shores around promoter regions, infer functional consequences for these metabolic perturbations. Although inhibition of TET enzymes is likely to be the main mechanism by which IDH mutations are linked to DNA hypermethylation in the cancers studied here, it is noteworthy that 2-HG accumulation also affects other dioxygenases, such as histone demethylases and prolyl hydroxylases, which could contribute to the observed phenotype. It is also important to note that although mutations in *TET* genes are frequently found in AML without *IDH1/2* mutations, they are extremely rare in CS and therefore such mutations are unlikely to account for a common pathogenic alternative to *IDH* mutation in this cancer. A separate study^21^ has revealed that of 90 CS analysed by either exome sequencing or a more targeted approach, only one tumour was found to contain a *TET2* mutation. This mutation has never been reported as functionally relevant in AML where these have been extensively studied^22^. This is supported by the fact that in LGG, where *IDH* mutations occur at the even higher frequency of 70–80%, no functional *TET2* mutations have been reported^23^.

The shared *IDH* mutation-correlated hypermethylation phenotype in AML, LGG, CC and now also in CS suggests that the same biological processes are likely to be affected in all four cancer types. Indeed, we identified that the RAR activation pathway is independently targeted in the above four malignancies; RAR signalling is often affected early in carcinogenesis^24^, suggesting an important role in tumour development. Moreover, retinoids have been shown to inhibit growth in various cancers, such as skin, bladder, kidney, prostate, and breast^25^, but even closely related cancers display unique targets and mechanisms of action for retinoids^25^, further supporting our finding of tissue-specific effectors in this shared pathway. Finally, Retinol-binding protein 1, a downstream target of RAR, has recently been shown to become hypermethylated following knock-in of a mt *IDH1* gene into a cancer cell line^26^ and we have confirmed hypermethylation of the Retinol-binding protein 1 promoter and associated downregulation of gene expression in *IDH* mt CS.

As TET is a key enzyme in the active demethylation pathway, it can be expected (at least in healthy cells) to be under tissue-specific regulation, and disruption of its function in cancer cells should therefore result in patterns of hypermethylation unique to its cell type of origin. Indeed, there is evidence for the existence of cellular memory from stem cell research^27^ and our meta-analysis of the differential methylation profiles in these four cancers (originating from different cells types) suggests that genes that are only affected in one cancer type are principally involved in functions and pathways specific to that cell type. Although the TET oxygenases (except TET2) are predicted to contain a CXXC domain that has been described to bind to CpG sites^9^, there is limited understanding of how they target specific genes, a prerequisite to maintaining the specificity of their regulatory function. In the absence of a targeting domain in TET itself, a likely mechanism is that a DNA-binding protein serves as an interaction partner for TET, such that the regulation of demethylation would be under the tissue-specific control of the interaction partner at the transcriptional or post-transcriptional level. We identified EBF1 as a potential candidate for this role at the transcriptional level through motif analysis of hypermethylated regions in four cancer types where TET function is impaired by increased levels of 2-HG; this hypothesis is supported by proportional enrichment for both EBF1 and TET2 at selected loci establishing co-localisation of the two proteins, and by co-immunoprecipitation of endogenous EBF1 with TET2 which demonstrates their interaction, either directly or indirectly, as part of a larger complex. This finding is also supported by the previously reported role of EBF1 in transcriptional regulation; for example, during B-cell differentiation, it has been associated with induction of *CD79a* promoter demethylation^28^, and EBF1 binding also correlates with histone modifications associated with transcriptional activation and poised chromatin^29^. In addition, depending on spatial or temporal contexts for instance, EBF1 could potentially be an interaction partner for other enzymes as well as TET2, such as dioxygenases or other chromatin modifiers, and the EBF1 interactome will need to be further studied to clarify the range of partners EBF1 interacts with. Our findings identify EBF1 as novel interaction partner of TET2, confirm *IDH* mt-mediated hypermethylation to be a recurrent phenomenon in unrelated types of cancer, and have identified tissue- and cancer- specific effectors as key drivers, and potential therapeutic targets.

## Methods

### Patient samples and mutation analysis

The material was obtained from the Stanmore Musculoskeletal Biobank, the approval for which was provided by the Cambridgeshire 1 Research Ethics Committee (Reference Number: 09/H0304/78).

*IDH* mutations were tested and validated by at least 2 of the following techniques including Sequenom MassARRAY, capillary sequencing, exome sequencing and a custom-made Taqman array^2^,^3^.

A total of 44 patient samples (21 *IDH*^*+/+*^ and 23 *IDH*^*+/*−^) and 4 technical controls were available for analysis; 8 were formalin-fixed paraffin-embedded (FFPE) tissue samples (6 *IDH* wt and 2 *IDH* mt) while the other 36 were fresh frozen (FF). An additional 6 *IDH* mt FF samples were used in the RainDrop-BSseq replication set, and a further 24 patient samples (10 wt and 14 mt) for the 450 K replication set.

### DNA extraction

DNA was extracted from FF tissue using the QIAamp DNA Mini Kit (QIAGEN) according to manufacturer’s instructions, and from FFPE with the QIAamp DNA FFPE Tissue Kit (QIAGEN) and REPLI-g FFPE Kit (QIAGEN)^30^.

### Methylation analysis

Bisulphite conversion of the DNA for methylation profiling was performed using the EZ DNA Methylation kit (Zymo Research) on 500 ng from FF samples and 1 μg from FFPE. Conversion efficiency was quantitatively assessed by quantitative PCR (qPCR).

The Illumina Infinium HumanMethylation450 BeadChips^12^ were processed as per manufacturer’s recommendations. Pyrosequencing validation was conducted using PyroMark Gold Q96 (QIAGEN) reagents and the PyroMark Q96 MD pyrosequencer as per manufacturer’s instructions. For the validation and replication using targeted microdroplet PCR bisulphite sequencing (RainDrop-BSseq), sample preparation and bisulphite conversion were carried out as described above. The parallel amplification of target loci was performed by RainDance Technologies (Lexington, MA, USA) and the subsequent sequencing by Illumina. The RainDance technology allows massively parallel amplification of specific DNA fragments by conducting PCR reactions in pico litre droplets on integrated microfluidic chips. The produced library (one for each sample) was then separately subjected to a second round of PCR to incorporate the sequencing indices. The libraries for all samples were pooled and sequenced on the Illumina MiSeq. For a more efficient comparison between RainDrop-BSseq and 450 K, we assigned each methylation score to bins of 20% increment in methylation, and considered those between 80–100% to be methylated and those between 0–20% to be unmethylated. These methylation states were then compared across platforms. A summary table of the results is available as Supplementary Table S1.

### ChIP-qPCR analysis

Automated ChIP was performed using the Auto-ChIP Kit (Diagenode) and the SX-8G IP-Star (Diagenode) according to the manufacturer’s instructions. 3 μg and 7 μg of TET2 (sc-136926; Santa Cruz) and EBF1 (clone 1G8, Abnova) antibodies, respectively, were used in each reaction. The SW1353 CS cell line used was obtained from ATCC (HTB-94). The target regions and negative control region are referred to here by the nearest gene; qPCR primers for these sites were designed using NCBI Primer Blast and manufactured by Sigma-Aldrich: CCND2:F: 5′-GTTTCTGCTCGAGGATCACA-3′, R:5′-GGGAGAGGTGGGTATTAGGA-3′, FABP3:F: 5′-CCTGGGGCTTCCTATTTCG-3′, R: 5′-TGCCGCTTTAAATAGCCCTC-3′, FBRSL1:F: 5′-TACGCGCTGCATGAATCAAT-3′, R: 5′-CTGGTGGGGTTTTCTGAGC-3′, OOEP: F: 5′-TATGGTCGATGATGCTGGTG-3′, R: 5′-GGGTCTCTCAGTTCCTGCAC-3′. The OOEP primer set was used as negative control. Quantitative PCR was performed on the Applied Biosystems 7300. Enrichments were assessed using the ΔΔCt method, normalising qPCR results to both the mock IgG IP and the negative control region site.

### Co-Immunoprecipitation and western blot analysis

Immunoprecipitation of the complex involving TET2 and EBF1 was performed with 3 μg of anti-TET2 antibody (sc-136926, Santa Cruz Biotechnology) and protein A sepharose in cell lysates of SW1353 cells with 5% of the lysate taken as input control before IP (lysis buffer: 30 mM HEPES, 20 mM β-glycerophosphate, 20 mM KCl, 1 mM ethylene glycol tetraacetic acid (EGTA), 2 mM NaF, 1 mM Na_3_VO_4_, 1% TX100, 1 mM benzamidine, 4 μM leupeptin, 5 mM PMSF, 1 mM DTT at pH 7.4). Immune complexes were washed three times with wash buffer (20 mM Tris pH 8.0, 1 M NaCl, 10% glycerol, 1% NP-40, 5 mN EDTA pH 8.0, 0.5 mM EGTA pH 8.0, 50 mM NaF, 20 mM β-glycerophosphate 1 mM Na3VO4), before subsequent detection of EBF1 and TET2 in the precipitate by western blotting using 1:500 and 1:1,000 dilutions of EBF1 (Abnova, H00001879-M02) and TET2 (Abcam, ab94580) antibodies, respectively, with 5% milk as blocking agent in TBST (TBS with 0.1% Tween 20). Secondary antibodies from GE Healthcare were used at 1:5,000 dilutions. See Supplementary Fig. S5 for full western blots.

### Statistical analysis

The raw output from the 450 K BeadChips was processed using GenomeStudio software (Illumina). Raw data is available from GEO (accession number GSE40853). The non-normalised and non-background corrected data and array annotation were exported as text files from GenomeStudio and all subsequent analysis was performed using the R statistical software v2.15.0 ( http://www.R-project.org) with R packages^31^,^32^,^33^,^34^ and custom scripts. Quality control of the data resulted in removal of samples showing reduced coverage, and any probes that did not pass a detection *P*-value threshold of 0.01 across all samples; after removal of technical replicates and control samples, a data set of 27 samples (12 *IDH*^*+/+*^ and 15 *IDH*^*+/*−^) and 472,655 probes were available for analysis.

A principal component analysis^35^ of the data was performed to identify the principal components of variation. Unsupervised consensus clustering was conducted on the top probes selected using a median absolute deviation (MAD) estimator, which provides a more robust measure of variance than standard deviation. We selected the top 150 most variable positions (MVPs) corresponding to a lower-end threshold of MAD=0.5. Thus, these selected probes show substantial variance with methylation differences across many samples in the order of 50% methylation changes. We note that we also performed consensus clustering on more MVPs by lowering the MAD threshold to include 300 and 500 probes, with identical results, demonstrating robustness to the choice of threshold.

A Wilcoxon rank-sum test was used for supervised analysis; *P*-values obtained from the latter were adjusted for multiple testing (Benjamini–Hochberg^36^) and only probes with *P*-value≤0.001 were used in the clustering. A further filter of absolute (Δ (medianβ)) ≥0.35 was used to compensate for the Wilcoxon rank-sum test not taking into account absolute difference in methylation between the groups, and to narrow down our search to differences with higher potential for functional effect.

The MVPs used to separate the validation sample sets (*n*=24, 10 wt and 14 mt) were selected based on the same method used for the filtering of MVPs in the initial data set, specifically ordering them by: (1) increasing adjusted *P*-value and then (2) decreasing absolute median difference between the mt and wt groups.

The statistical significance of the observed percentage enrichments for genomic and epigenomic features among the 3,057 MVPs was calculated on the basis of 1000 repetitions of a random selection of 3057 probes from the overall probe set (472,655 probes) used in the analysis. The aforementioned features correspond to the official annotation of the 450 K BeadChips, and were extracted using GenomeStudio.

Raw sequencing reads from the microdroplet PCR were trimmed to 60 bp as recommended by Krueger *et al*.^37^, and fastq_quality_trimmer from the fastx toolkit ( http://hannonlab.cshl.edu/fastx_toolkit/index.html) was used to trim lower quality bases from the ends of sequence reads (threshold set at 30); reads were trimmed down to a minimum length of 20 bp.The alignment was conducted using Bismark^38^, specifically designed for mapping bisulphite converted sequence reads. Finally, methylation states were determined using the Bismark methylation_extractor and custom perl scripts. CpG sites covered by 10 sequencing reads or more and with methylation scores between 0–20% (unmethylated) or 80–100%(methylated) were selected.

### Meta-analysis

For the meta-analysis, we used the published list of differentially methylated genes for AML (*n*=398, 347 wt and 51 mt), significantly differentially methylated genes (Wilcoxon *P*-value≤0.001, |Δß|≥0.35) for LGG (*n*=81, 32 wt and 49 mt) and CC (*n*=50, 31 wt and 19 mt) and the data reported here for CS, with a further restriction to sites found in gene promoters and CpG islands/shores. The functional analysis identified the biological functions that were most significant to the data set. Right‐tailed Fisher’s exact test was used to calculate a *P*‐value determining the probability that each biological function assigned to that data set is due to chance alone. This *P*-value was further adjusted (Benjamini–Hochberg^36^) for multiple testing. Canonical pathway analysis identified the pathways from the IPA library of canonical pathways that were most significant to the data set. Molecules from the data set that were associated with a canonical pathway in the Ingenuity Knowledge Base were considered for the analysis. Fisher’s exact test was used to calculate a *P*‐value determining the probability that the association between the genes in the data set and the canonical pathway is explained by chance alone. This *P*-value was further adjusted (Benjamini–Hochberg^36^) for multiple testing. The motif analysis was conducted using the online MEME suite of tools^17^: FASTA sequences were downloaded from the UCSC Genome Browser, and used for input in the MEME-ChIP tool of the MEME suite; parameters were set to default except for the number of repetitions (set to ‘Any number of repetitions’), motif width (min=4, max=15), and maximum number of motifs to find (20).

## Author contributions

A.M.F., M.T.R. and S.B. conceived the study. A.M.F., R.T., M.F.A., D.B. and G.J. provided and M.E. and D.H. prepared the tumour samples. P.G. performed the experiments and analyses. G.A.W., A.F. and A.E.T. contributed to the bioinformatic and statistical analyses. V.G. and A.H. provided materials and advice on the co-IP and western blot experiments. A.M.F., M.T.R. and S.B. supervised the project. P.G., A.M.F. and S.B. wrote the manuscript with contributions from all co-authors.

## Additional information

**Accession codes:** Sequencing data have been deposited in the Gene Expression Omnibus under series expression code GSE40853.

**How to cite this article:** Guilhamon, P. *et al*. Meta-analysis of IDH-mutant cancers identifies EBF1 as an interaction partner for TET2. *Nat. Commun.* 4:2166 doi: 10.1038/ncomms3166 (2013).

## Supplementary Material

Supplementary Information Supplementary Figures S1-S5 and Supplementary Tables S1-S2
