# Supplementary material for: Meta-analysis of IDH-mutant cancers identifies EBF1 as an interaction partner for TET2
Source: Nat Commun. 2013 Jul 18;4:2166. doi: 10.1038/ncomms3166 (PMC3759038; doi:10.1038/ncomms3166)
Supplement: Supplementary Information — Supplementary Figures S1-S5 and Supplementary Tables S1-S2 [file ncomms3166-s1.pdf]

Meta-analysis of IDH-mutant cancers identifies EBF1 as a novel interaction partner for TET2

Paul Guilhamon[1], Malihe Eskandarpour[2], Dina Halai[3], Gareth A. Wilson[1], Andrew Feber[1], Andrew E. Teschendorff[4], Valenti Gomez[5], Alexander Hergovich[5], Roberto Tirabosco[3], M. Fernanda Amary[3], Daniel Baumhoer[6], Gernot Jundt[6], Mark T. Ross[7], Adrienne M. Flanagan[2,3], Stephan Beck[1*]

[1]Medical Genomics, UCL Cancer Institute, University College London, London, UK

[2]Genetics and Cell Biology of Sarcoma, UCL Cancer Institute, University College London, London, UK

[3]Department of Histopathology, Royal National Orthopaedic Hospital NHS Trust, Stanmore, Middlesex, UK

[4]Statisical Cancer Genomics, UCL Cancer Institute, University College London, London, UK

[5]Tumour Suppressor Signalling Networks, UCL Cancer Institute, University College London, UK

[6]Bone Tumor Reference Center at the Institute of Pathology, University Hospital Basel, Basel, Switzerland

[7]Illumina Cambridge Ltd., Chesterford Research Park, Little Chesterford, UK

*corresponding author: SB <s.beck@ucl.ac.uk>

SUPPLEMENTARY FIGURES

Supplementary Figure S1

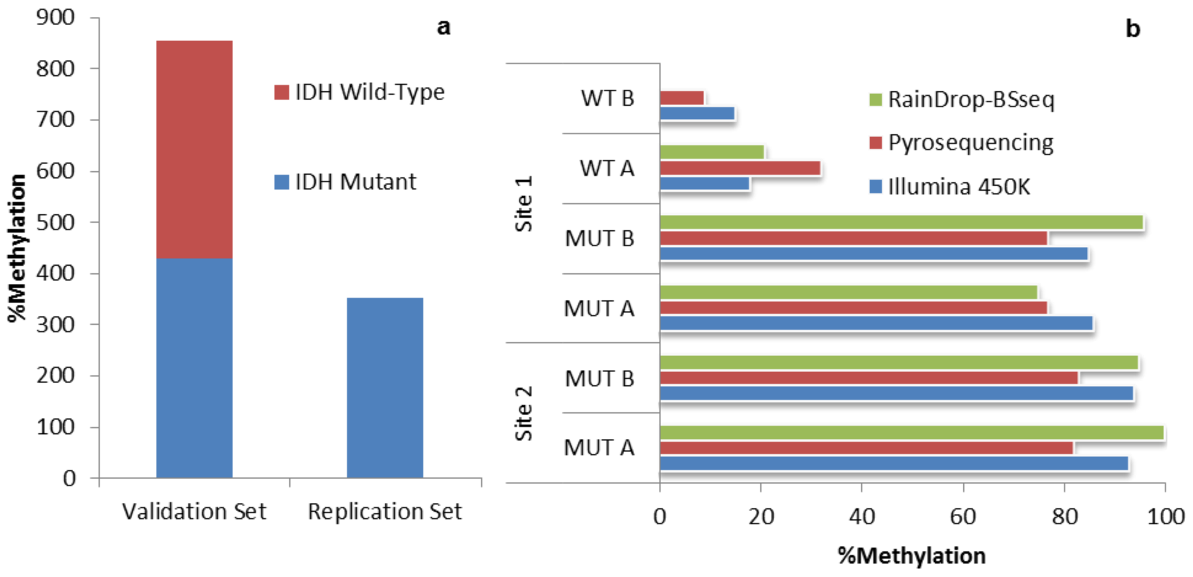

Supplementary Figure S1: High-throughput validation of MVPs identified with Infinium 450k BeadChips

a) Cumulative bar chart of MVPs validated by RainDrop-BSseq. Of the selected MVPs, 855 across 16 samples (11 *IDH* mt + 5 *IDH* wt) validated, representing 98.8% and 95.5% in the wt and mt groups, respectively. Bar chart of MVPs validated by RainDrop-BSseq in the replication set (n=6). Of the selected MVPs, 352 (94.3%) matched the values measured in the validation set.

b) Cross-platform validation for four MVPs at two different genomic sites. MVPs at the two sites represented a range of methylation levels (low, intermediate, high). All three methods produced similar results, with measurements at each site within 19% (max beta difference) of each other.

Supplementary Figure S2

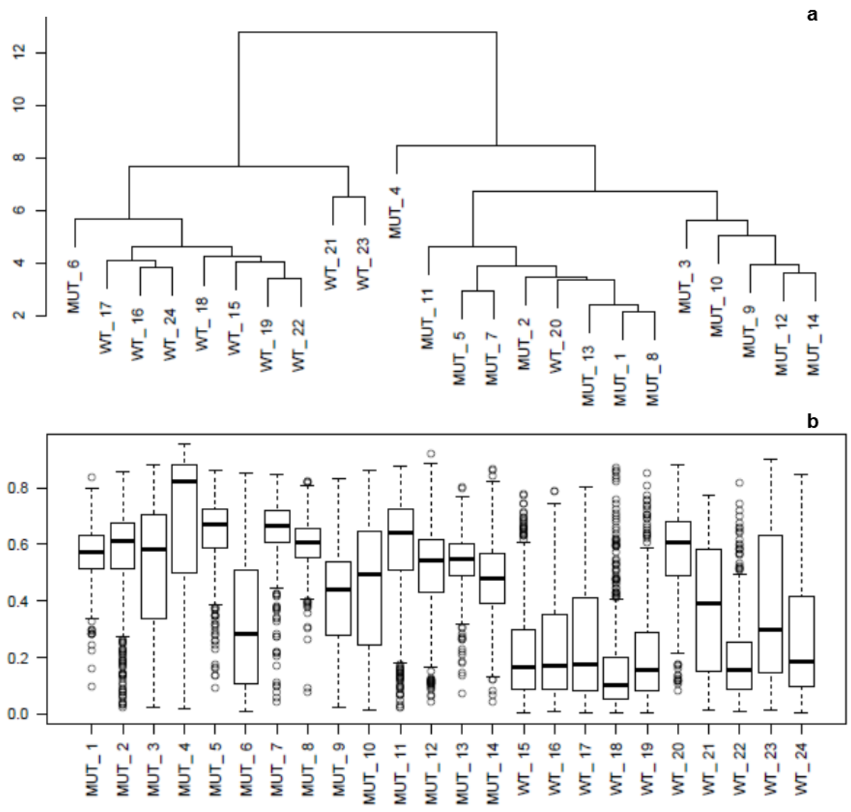

Supplementary Figure S2: Replication set with Infinium 450K BeadChips

a) Supervised hierarchical clustering of the 24 samples in the 450K replication set using the top 500 MVPs from the first cohort. The samples separate into two clusters, with 92% (22/24) of samples grouped in the correct cluster.

b) Box plot of β-values from the 24 samples in the 450K replication set using the top 500 MVPs from the first cohort. The mt sample group are clearly hypermethylated relative to the wt.

Supplementary Figure S3

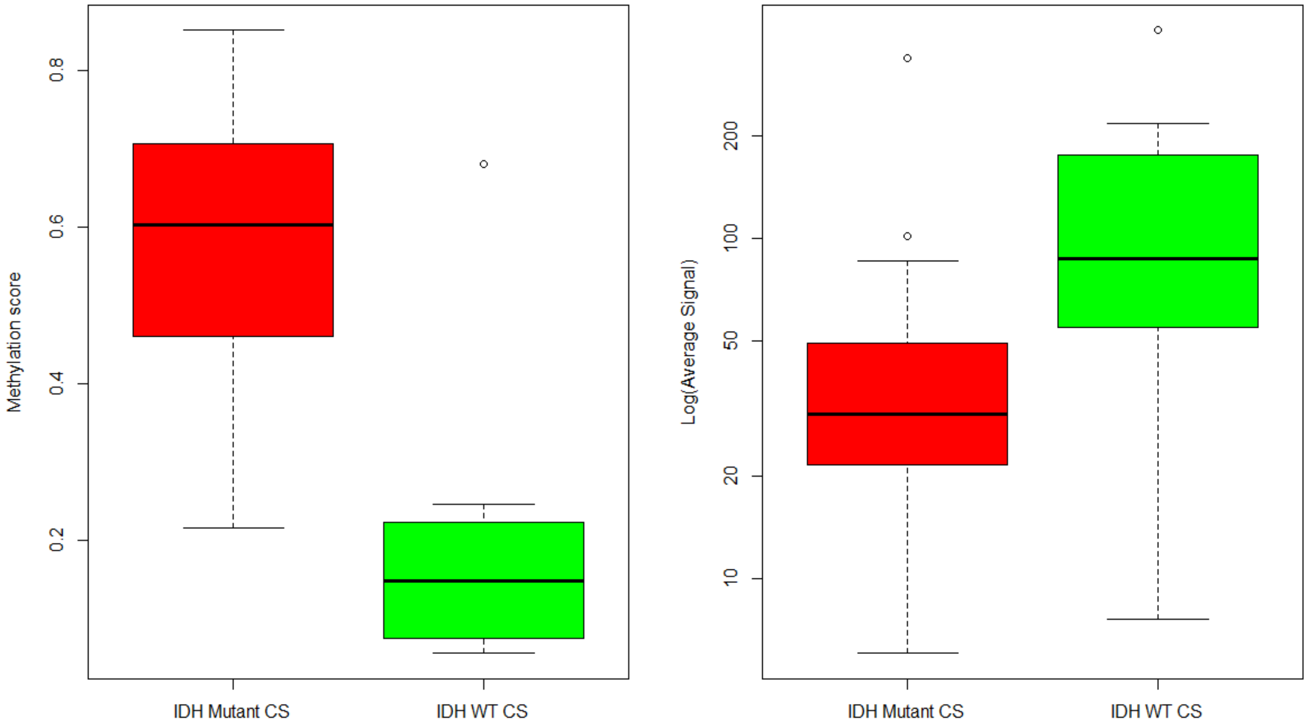

Supplementary Figure S3: RBP1 methylation and gene expression in CS

a) The RBP1 promoter is significantly hypermethylated (p-value=6.9×10^{-6}) in chondrosarcoma harbouring an IDH1 mutation compared to IDH1 wt.

b) RBP1 gene expression is also significantly (p-value=0.018) downregulated in mt samples.

Supplementary Figure S4

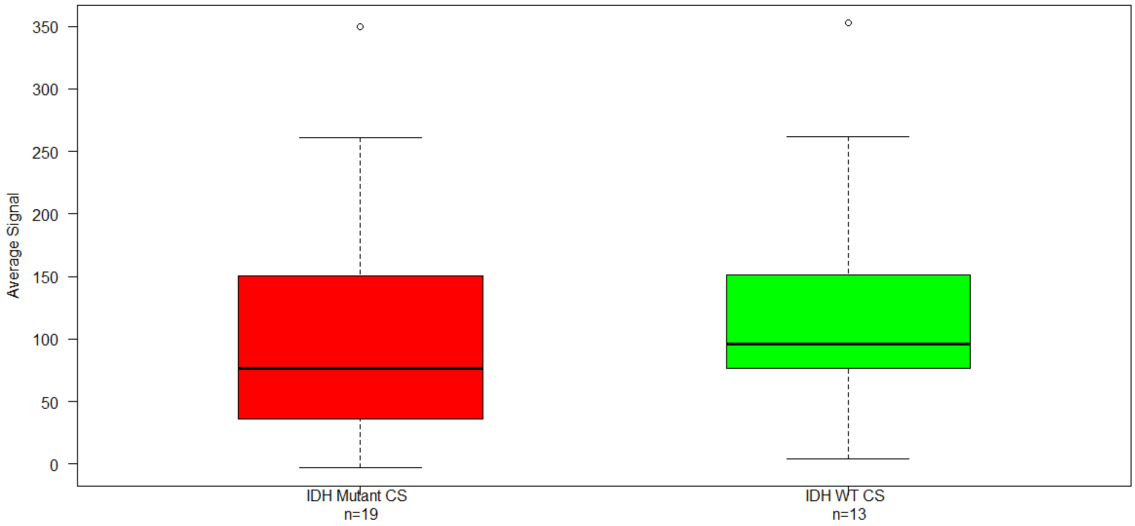

Supplementary Figure S4: EBF1 gene expression in IDH mt and IDH wt chondrosarcoma

EBF1 gene expression was assessed in a panel of 32 CS samples (19 mt and 13 wt) using gene expression microarrays. Both sample groups display similar levels of expression.

Supplementary Figure S5

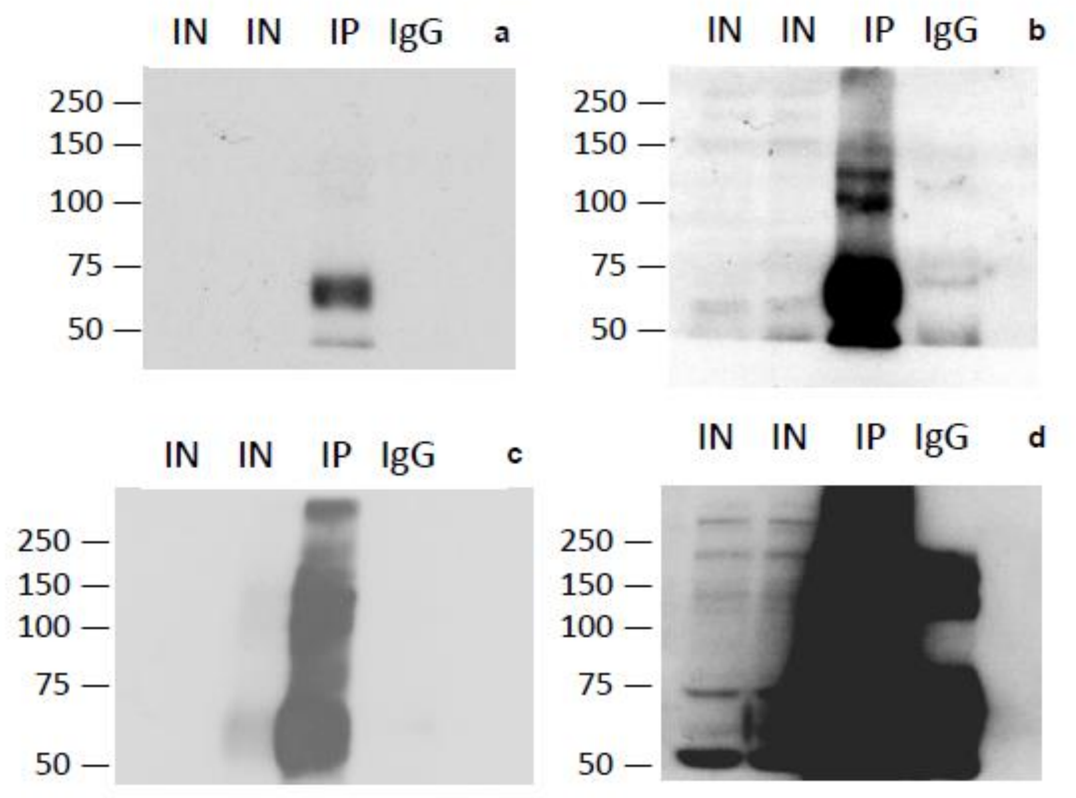

Supplementary Figure S5: EBF1 and TET2 Western Blots

a) Blot against EBF1; 1 min exposure to visualise IP and control.

b) Blot against EBF1; 1h exposure to visualise input samples.

c) Blot against TET2; 5sec exposure to visualise IP and control.

d) Blot against TET2; 1 min exposure to visualise input samples.

Molecular weights are shown in kDa.

SUPPLEMENTARY TABLES

Supplementary Table S1

	Validation Set			
	**n**	**CpGs Validated**	**CpGs not validated**	**%Validated**
**Wild-Type**	5	426	5	98.8
**Mutant**	11	429	20	95.5
	**Replication Set**			
**Mutant**	6	352	21	94.3

Supplementary Table S1: High-throughput validation and replication of MVPs identified with Infinium 450k BeadChips

Of the selected MVPs, 855 across 16 samples (11 mt + 5 wt) validated, representing 98.8% and 95.5% in the in the wt and mt groups, respectively. Of the 371 MVPs assessed in the replication set (n=6), 352 (94.3%) matched the values measured in the validation set.

Supplementary Table S2

	IDH Mutant CS	IDH Wild-Type CS	p-value (BH)	SW1353
**CCND2**	0.6238	0.0185	1.92E-04	0.9764
**FABP3**	0.6331	0.0227	3.23E-04	0.9882
**FBRSL1**	0.6555	0.0276	1.49E-04	0.9840

Supplementary Table S2: Characteristics of selected EBF1 and TET2 target loci

Target sites share a significant hypermethylation in the CS mt cohort as compared to the wt samples, and associated high methylation levels in the SW1353 cell line used for functional validation.
